# Effectiveness of Delivering Dialectical Behavioral Therapy Techniques by Email in Patients With Borderline Personality Disorder: Nonrandomized Controlled Trial

**DOI:** 10.2196/27308

**Published:** 2021-04-30

**Authors:** Nazanin Alavi, Callum Stephenson, Margo Rivera

**Affiliations:** 1 Department of Psychiatry Queen's University Kingston, ON Canada; 2 Centre for Neuroscience Studies Faculty of Health Sciences Queen's University Kingston, ON Canada; 3 Personality Disorder Services Queen's University Kingston, ON Canada

**Keywords:** borderline personality disorder, treatment, psychotherapy, psychotherapy, dialectical behavioral therapy, barriers to treatment, mental health, online, internet, electronic

## Abstract

**Background:**

Borderline personality disorder is a debilitating and prevalent mental health disorder, with often inaccessible treatment options. Electronically delivered dialectical behavioral therapy could be an efficacious and more accessible intervention.

**Objective:**

We aimed to evaluate the efficacy of electronic delivery of dialectical behavioral therapy in the treatment of individuals with symptoms of borderline personality disorder.

**Methods:**

Study participants diagnosed with borderline personality disorder were offered either an email-based or in-person group format dialectical behavioral therapy skill-building program. During each session, participants were provided with both the material and feedback regarding their previous week’s homework. Electronically delivered dialectical behavioral therapy protocol and content were designed to mirror in-person content. Participants were assessed using the Self-Assessment Questionnaire (SAQ) and Difficulties in Emotion Regulation Scale (DERS).

**Results:**

There were significant increases in SAQ scores from pre- to posttreatment in the electronic delivery group (*F*_1,92_=69.32, *P*<.001) and in-person group (*F*_1,92_=60.97, *P*<.001). There were no significant differences observed between the groups at pre- and posttreatment for SAQ scores (*F*_1,92_=.05, *P*=.83). There were significant decreases in DERS scores observed between pre- and posttreatment in the electronic delivery group (*F*_1,91_=30.15, *P*<.001) and the in-person group (*F*_1,91_=58.18, *P*<.001). There were no significant differences observed between the groups for DERS scores pre- and posttreatment (*F*_1,91_=.24, *P*=.63). There was no significant difference in treatment efficacy observed between the 2 treatment arms (*P*<.001).

**Conclusions:**

Despite the proven efficacy of in-person dialectical behavioral therapy in the treatment of borderline personality disorder, there are barriers to receiving this treatment. With the prevalence of internet access continuing to rise globally, delivering dialectical behavioral therapy with email may provide a more accessible alternative to treatment for individuals with borderline personality disorder without sacrificing the quality of care.

**Trial Registration:**

ClinicalTrials.gov NCT04493580; https://clinicaltrials.gov/ct2/show/NCT04493580

## Introduction

The essential features of a personality disorder are an impairment in personality (self and interpersonal) functioning and the presence of pathological personality traits, including impairments in identity, self-direction, and interpersonal functioning [1]. Additionally, it is common for an individual with a personality disorder to present with numerous comorbid mental disorder diagnoses [2]. Moreover, it is well documented that the presence of a personality disorder negatively impacts the efficacy of treatment for other physical and mental disorders [1,3,4].

Borderline personality disorder is a serious psychiatric disorder with a prevalence of approximately 1% to 2% in the general population [5]. Borderline personality disorder is characterized by a pervasive pattern of mental instability in the areas of affect regulation, impulse control, interpersonal relationships, and self-image. Clinical signs of borderline personality disorder include emotional dysregulation, impulsive aggression, repeated intentional self-injury, and chronic suicidal tendencies and ideation [6].

Dialectical behavior therapy (DBT) is one of the most efficacious and commonly utilized modalities for the treatment of borderline personality disorder [7]. DBT is structured with weekly group meetings where participants can learn about and develop coping skills. This learning and development are done using an evidence-based manualized curriculum that encompasses the topics of mindfulness, interpersonal relationships, emotion regulation, and distress tolerance [7,8].

Although various psychotherapy modalities have been proven to be effective in treating borderline personality disorder, they are often not used due to limited resources and lack of accessibility for patients. The causes of inaccessibility to treatment can be broken into 2 categories—practical, such as long waitlists, rural or remote living situations where treatment is not available, and lack of transportation, and psychological, wherein individuals with borderline personality disorder are more resistant to the idea of participating in group therapy than individuals with other mental disorders [9]. A commonly reported reason for avoiding in-person psychotherapy is to avoid the stigma surrounding mental health [10]. Due to these factors, while potentially effective, in-person DBT does not appear to be an ideal treatment modality for individuals with borderline personality disorder, and the issues mentioned must be addressed in the hopes of developing a more accessible treatment option.

A viable treatment delivery method may be using the internet. Internet usage is increasing exponentially, with over 2.5 billion people globally using the internet [11]. Moreover, there was a reported increase of 56% in internet users globally from 2000 to 2012 [11]. Currently, approximately 41% of households worldwide can connect to the internet, and approximately 37% of women and 41% of men use the internet [11]. For individuals in high-income economies, internet use has become an integral part of daily life. Even within middle- and low-income countries, internet usage continues to increase [11]. With higher speeds, more affordable access, and an increasing user base, there is a growing demand for more robust and sophisticated technologies and applications on the internet [11].

Given the culture shift with respect to internet communication, it is not surprising that there has been rapid growth in recent years in the research, development, and use of internet-based psychotherapeutic interventions. To address the issue of the abovementioned barriers to access, the use of internet-based psychotherapy, which is clinically effective, has emerged as a solution [12]. In-person and live participation in psychotherapy is no longer the exclusive treatment delivery route for individuals to address their mental health needs. Fortunately, research has shown that electronically delivered cognitive behavioral therapy (e-CBT) is a cost-effective and easily accessible treatment modality for a wide variety of mental health disorders [13,14]. Furthermore, it has been suggested that internet-based psychotherapy can increase treatment adherence and yield high treatment satisfaction among patients while offering results comparable to those offered by in-person psychotherapy. For the treatment of depression, e-CBT programs have demonstrated considerable efficacy and have been increasingly utilized to enhance access to care for individuals [15,16]. Although there has been a large amount of research on the efficacy of e-CBT in the treatment of mental health disorders, to date, no study has examined the efficacy of electronically delivered DBT (e-DBT) skill-building programs for treating individuals with borderline personality disorder.

We aimed to add to the literature by creating and offering an email-based DBT skill-building program as an alternative treatment modality for individuals with borderline personality disorder who were referred to participate in in-person DBT. Additionally, we aimed to explore the efficacy and accessibility of e-DBT compared to in-person DBT.

## Methods

### Dialectical Behavioral Therapy

Since 1995, the Personality Disorders Service in Kingston, Ontario, Canada, has developed psychotherapeutic programs that have integrated a range of modalities for the treatment of individuals with borderline personality disorder [17,18]. Currently, several therapy groups are offered, including a weekly skill-building group structured to offer the basic DBT curriculum, titled *Managing Powerful Emotions*. This curriculum includes mindfulness, distress tolerance, emotion regulation, and interpersonal effectiveness and has been offered since 2000 as a first-line treatment for individuals diagnosed with borderline personality disorder.

The Personality Disorders Service offers more advanced therapy groups for individuals who have completed the Managing Powerful Emotions program and wish to continue seeking treatment modalities. One of the most intensive therapy programs offered is the Chrysalis Day Treatment Program [17,18]. To participate in the Chrysalis Day Treatment Program, an individual must progress through 2 prior phases: (1) Managing Powerful Emotions and (2) psychotherapy groups incorporating DBT skill-building. The Chrysalis Day Treatment Program is an intensive day treatment program that integrates DBT skill-building, psychodynamic psychotherapy, and a range of other group therapy modalities. This integrated form of psychotherapy is extremely effective, particularly in individuals with more severe and prolonged symptomology and trauma histories [18].

### Recruitment

Individuals who were referred to the Personality Disorders Service in Kingston, Ontario, Canada (after confirmation of diagnosis of borderline personality disorder by a psychiatrist in the Department of Psychiatry at Queen’s University) were offered the opportunity to select either the in-person DBT skill-building program or an email format of the program. Inclusion criteria were being between the ages of 18 and 65 years at study inception and a diagnosis of borderline personality disorder according to the Diagnostic and Statistical Manual of Mental Disorders Fifth Edition guidelines. Moreover, participants were required to have the competency to consent and participate, the ability to speak and read English, and to have consistent and reliable access to the internet. Participants were excluded from the study if they were experiencing acute hypomanic or manic episodes, were experiencing acute psychosis, had severe alcohol or substance use disorders, or were currently receiving DBT.

Individuals who were referred to the program were provided with an information sheet with details of the study and the comparative effectiveness of online and in-person treatment. Individuals were asked to give informed consent (ie, sign a letter of consent) to participate in the study. The in-person treatment group served as a control group.

### Measurement Scales

All participants were required to complete questionnaires at baseline, at the end of week 7, and after the completion of the treatment program. These questionnaires included the Self-Assessment Questionnaire (SAQ) and the Difficulties in Emotion Regulation Scale (DERS) [19]. The DERS is a self-report tool designed to obtain an overall measure of the difficulty respondents have with various aspects of emotion regulation. The DERS provides an overall score of difficulties with emotion regulation as well as an assessment of each of the following 6 specific factors related to emotion dysregulation: nonacceptance (nonacceptance of emotional responses), goals (difficulty engaging in goal-oriented behaviors), impulse (difficulty controlling impulses), awareness (lack of emotional awareness), strategies (lack of access to emotion regulation strategies), and clarity (lack of emotional clarity).

Participants were informed that both in-person and e-DBT treatment programs were created with the intent of helping them to learn useful skills and strategies for managing emotions and behaviors and that it was not to be used as a crisis service. Participants of the electronically delivered program were informed that their therapist would read their emails once a week and would not be able to respond to crises, such as acute suicidal ideation or intent. Participants were informed that, in the case of an emergency, they should either go to their local emergency department or call emergency services or their local crisis line.

### Therapy Programs

The study protocol was registered (NCT04493580). Both programs had a duration of 15 weeks, with 1 DBT session per week. In the e-DBT group, participants were individually emailed approximately 30 to 40 PowerPoint slides (Microsoft Inc) each week that they were to complete. These slides included general information on a particular topic (Table 1), an overview of skills related to the topics being covered, and homework sheets to be completed and returned to their therapists. The team of therapists involved in care delivery were psychiatry residents, psychologists, and registered nurses who also facilitated the in-person groups. All content and the format of the e-DBT program were designed to directly correspond with those of the in-person group.

Participants in the e-DBT program were asked to email their homework sheets back to their therapist by a specific day each week. The following day, the therapist would review the homework and email the participant individualized feedback regarding their homework along with the following week’s homework and slides. To be eligible to receive the following week's materials, participants were required to email their homework before the set deadline. If the homework was not submitted before the deadline, a reminder email was sent. If a participant missed more than 2 sessions, they were removed from the study.

**Table 1:** Managing Powerful Emotions sessions.

Week	Content
1	Goals, Accepting Reality, Willingness vs. Willfulness, Distress Tolerance Box
2	Crisis Survival Strategies – Distract
3	Crisis Survival Strategies – Self-Soothe
4	Crisis Survival Strategies – Improve the Moment
5	Crisis Survival Strategies – Pros and Cons
6	Skills for Accepting Life as it is in the Moment – Observing Your Breath
7	Skills for Accepting Life as it is in the Moment – Half-Smiling Exercises
8	Skills for Accepting Life as it is in the Moment – Awareness Exercises
9	Distress Tolerance Box Due / Emotion Regulation
10	Myths About Emotion
11	Model for Describing Emotion, Emotion Sheet
12	Functions of Emotion
13	Reducing Vulnerability to Painful Emotions, Increasing Pleasant Events
14	Acting Opposite to Action Urge
15	Your Opinions

### Analysis

To determine whether there was a significant change in functioning or level of symptomatology pre- to posttreatment, *t* tests and mixed-model analysis of variance 2 (e-DBT, in-person) ×2 (pretreatment, posttreatment) with Bonferroni correction were used. Between group and within-group differences at baseline were assessed using *t* tests.

### Ethical Considerations and Confidentiality

Only individuals involved in the direct care of participants had access to their information. Data regarding study variables were entered anonymously into a database separate from clinical files using anonymous participant identification numbers. This study was approved by the Queen’s University Research and Ethics Board (TRAQ 6007697; PSIY-391-13).

## Results

### Participants

**Figure 1:**
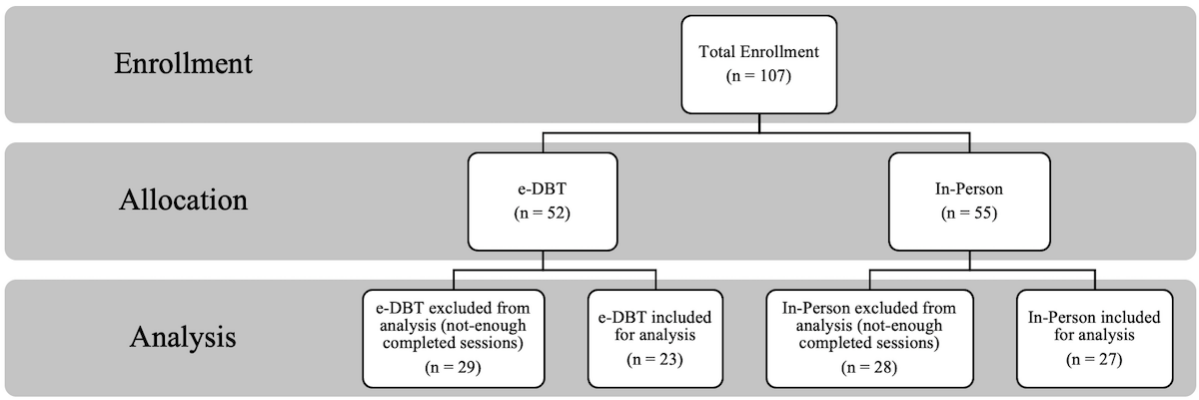
Participant enrollment, allocation, and analysis process. e-DBT: electronically delivered dialectical behavioral therapy.

**Figure 2:**
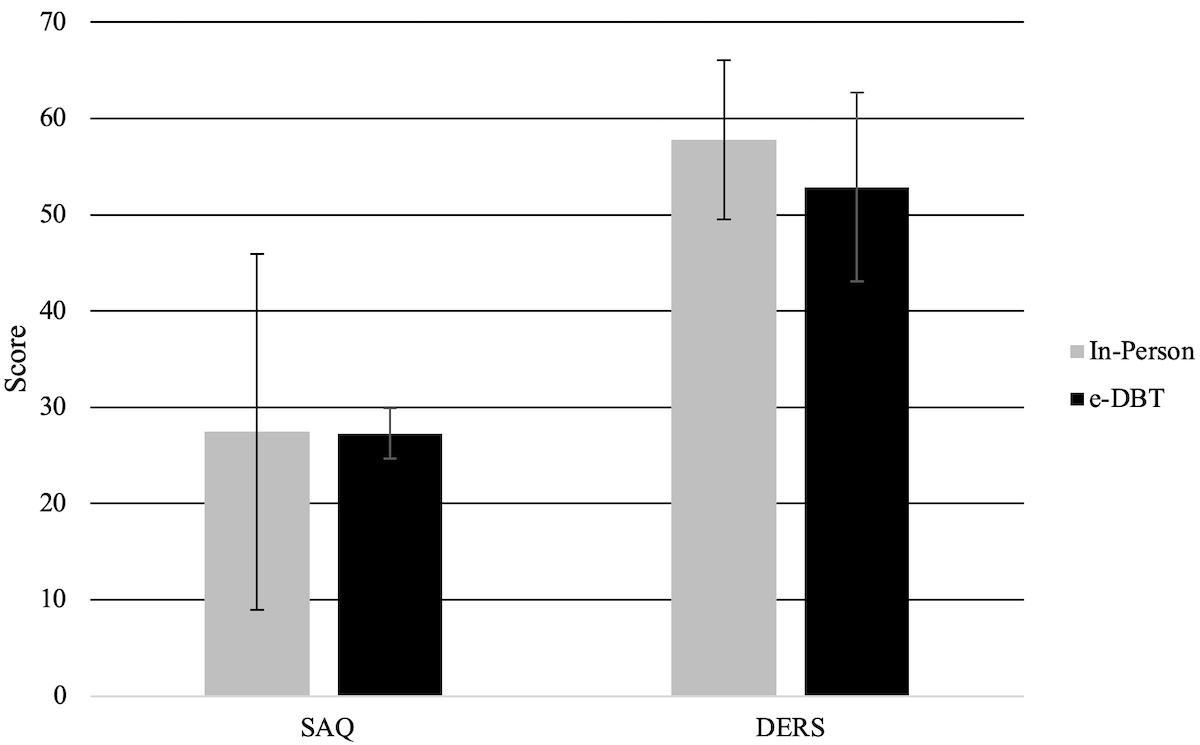
Prior to treatment, there were no significant differences between the 2 groups in Self-Assessment Questionnaire (SAQ) scores and Difficulties in Emotion Regulation Scale (DERS) scores. e-DBT: electronically delivered dialectical behavioral therapy.

Of 107 individuals recruited for the study, 52 elected to take part in the e-DBT group (male n=10, female n=42), and 55 elected to be in the in-person group (male n=14, female n=41) (Figure 1). At baseline, there were no significant differences between the 2 groups in SAQ (e-DBT: mean 27.29, SD 6.36; in-person: mean 27.45, SD 48.00; *t*_105_=–.11, *P*=.91) or DERS scores (e-DBT: mean 52.88, SD 23.45; in-person: mean 57.81, SD 21.51; *t*_104_=–1.13, *P*=.26) (Figure 2). Of the participants in the e-DBT group, 23 completed all therapy sessions, with those who did not complete all sessions completing between 2 and 13 sessions (mean 8.83, SD 3.30). Of the participants in the in-person group, 27 completed all therapy sessions, with those not completing all sessions completing between 1 and 14 sessions (mean 4.29, SD 3.46).

### SAQ Scores

**Figure 3:**
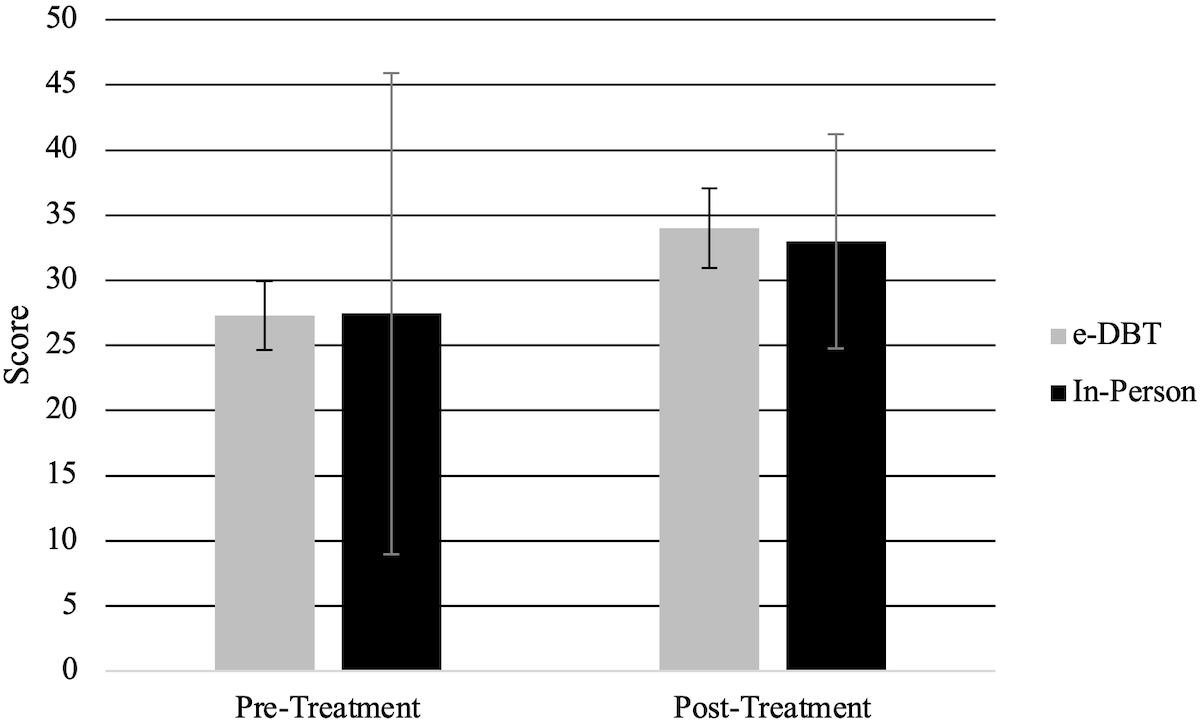
Self-Assessment Questionnaire scores at baseline (pre) and 15 weeks (post). e-DBT: electronically delivered dialectical behavioral therapy.

SAQ scores (Figure 3) were significantly different between pre- and posttreatment (*F*_1,92_=130.23, *P*<.001), with pretreatment scores (mean 27.57, SD 7.25) being significantly lower than posttreatment scores (mean 33.28, SD 7.66). Within the groups, significant increases in SAQ scores from pre- to posttreatment in the e-DBT group (*F*_1,92_=69.32, *P*<.001) and in-person group (*F*_1,92_=60.97, *P*<.001) were observed. There was no significant difference between the groups between pre- and posttreatment for SAQ scores (*F*_1,92_=0.05, *P*=.83).

### DERS Scores

**Figure 4:**
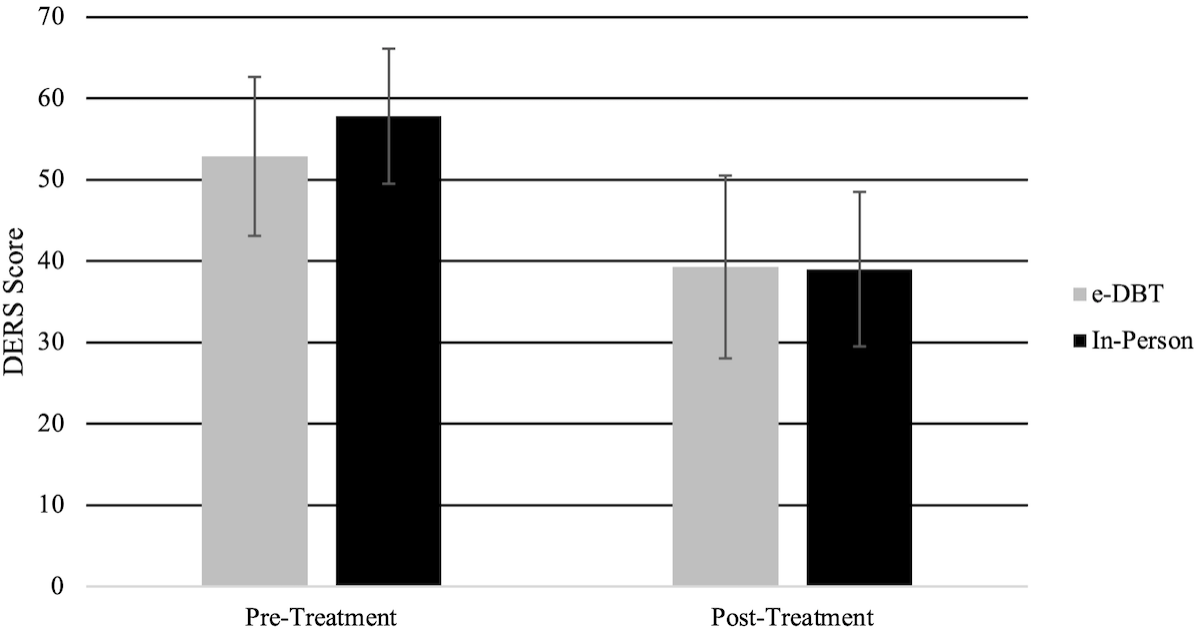
Difficulties in Emotion Regulation Scale (DERS) scores at baseline and 15 weeks. e-DBT: electronically delivered dialectical behavioral therapy.

DERS scores (Figure 4) were significantly different between pre- and posttreatment (*F*_1,91_=85.90, *P*<.001), with pretreatment scores (mean 54.68, SD 22.80) being significantly higher than posttreatment scores (mean 38.78, SD 23.78). Within the groups, there were significant decreases in DERS scores between pre- and posttreatment in the e-DBT group (*F*_1,91_=30.15, *P*<.001) and the in-person group (*F*_1,91_=58.18, *P*<.001). There was no significant difference between the groups between pre- and posttreatment for DERS scores (*F*_1,91_=0.24, *P*=.63).

### Nonacceptance Subscale Scores

The nonacceptance subscale scores significantly differed between pre- and posttreatment (*F*_1,91_=249.86, *P*<.001), with pretreatment scores (mean 19.70, SD 5.69) being significantly higher than posttreatment scores (mean 13.86, SD 5.26). Within the groups, there were significant decreases in nonacceptance scores between pre- and posttreatment in the e-DBT group (*F*_1,91_=123.98, *P*<.001) and the in-person group (*F*_1,91_=125.90, *P*<.001). There was no significant difference between the groups between pre- and posttreatment (*F*_1,91_=.11, *P*=.74).

### Goals Subscale Scores

The goals subscale scores were significantly different between pre- and posttreatment (*F*_1,92_=117.10, *P*<.001), with pretreatment scores (mean 14.15, SD 4.54) being significantly higher than posttreatment scores (mean 10.97, SD 4.71). Within the groups, there were significant decreases in goals scores between pre- and posttreatment in the e-DBT group (*F*_1,92_=48.96, *P*<.001) and the in-person group (*F*_1,92_=69.25, *P*<.001), with no significant difference between the groups between pre- and posttreatment (*F*_1,92_=.001, *P*=.97).

### Impulse Subscale Scores

The impulse subscale scores were significantly different between pre- and posttreatment (*F*_1,79_=131.33, *P*<.001), with pretreatment scores (mean 12.78, SD 7.74) being significantly higher than posttreatment scores (mean 8.36, SD 5.08). Within the groups, there were significant decreases in impulse scores between pre- and posttreatment in the e-DBT group (*F*_1,79_=69.01, *P*<.001) and the in-person group (*F*_1,79_=63.38, *P*<.001), with no significant difference between the groups between pre- and posttreatment (*F*_1,79_=0.005, *P*=.94).

### Awareness Subscale Scores

The awareness subscale scores were significantly different between pre- and posttreatment (*F*_1,76_=27.31, *P*<.001), with pretreatment scores (mean –17.22, SD 5.80) being significantly higher than posttreatment scores (mean –19.69, SD 6.92). Within the in-person group, there were significant differences between pre- and posttreatment scores (*F*_1,76_=32.20, *P*<.001); however, within the e-DBT group, there was no difference between pre- and posttreatment scores, (*F*_1,76_=1.84, *P*=.18). There was no significant difference between the groups between pre- and posttreatment (*F*_1,76_=.71, *P*=.40).

### Strategies Subscale Scores

The strategies subscale scores were significantly different between pre- and posttreatment (*F*_1,91_=171.73, *P*<.001), with pretreatment scores (mean 21.73, SD 7.12) significantly higher than posttreatment scores (mean 15.62, SD 6.90). Within the groups, there were significant decreases in strategies scores between pre- and posttreatment in the e-DBT group (*F*_1,91_=79.10, *P*<.001) and the in-person group (*F*_1,91_=93.00, *P*<.001), with no significant difference between the groups between pre- and posttreatment (*F*_1,91_=0.013, *P*=.91).

### Clarity Subscale Scores

The clarity subscale scores were significantly different between pre- and posttreatment (*F*_1,75_=121.03, *P*<.001), with pretreatment scores (mean 2.69, SD 4.00) significantly higher than posttreatment scores (mean –1.18, SD 3.65). Within the groups, there were significant decreases in clarity scores between pre- and posttreatment in the e-DBT group (*F*_1,91_=78.85, *P*<.001) and the in-person group (*F*_1,91_=47.65, *P*<.001), with no significant difference between the groups between pre- and posttreatment (*F*_1,75_=0.005, *P*=.94).

## Discussion

### General

DBT is a form of psychotherapy that has been proven to be an efficacious treatment modality for addressing various mental health disorders in several controlled research studies [7]. DBT is particularly effective in reducing the incidence of suicidal and self-injurious behaviors and the frequency of acute hospitalizations in individuals diagnosed with borderline personality disorder [20].

### Significance and Impact

Many individuals with borderline personality disorder are resistant to taking part in in-person group psychotherapy, a core aspect of DBT [20,21]. Additionally, there are many other psychological, social, geographical, and systemic barriers to utilizing DBT as a treatment for mental health disorders [9]. The demand for DBT often exceeds the resources, leaving many individuals with serious and life-threatening (in some cases) problems, on waitlists for evidence-based care. Therefore, it is an unequivocal public health need to overcome these barriers through alternative methods of care delivery. With internet use increasing globally, offering internet-based DBT skill-building groups through email (e-DBT) could be a viable treatment option that could help the health care system meet the demand for therapy. The ability to reduce treatment costs while offering comparable quality of care with more efficient utilization of medical personnel can be significant to the health care system. Among other benefits, e-DBT would allow for greater treatment accessibility to participants, as well as being more time-efficient for clinicians without having to sacrifice the quality of care. Moreover, e-DBT would allow for relatively simple modifications in the future when addressing language and cultural barriers to therapy. Additionally, e-DBT can provide a much-needed service to individuals located in geographically isolated areas.

Our results suggest that an e-DBT skill-training program delivered via email could be a viable treatment delivery modality for addressing symptoms in individuals diagnosed with borderline personality disorder. There were no significant differences observed in SAQ or DERS scores between the e-DBT and in-person groups both pre- and posttreatment (*P*<.001). This suggests that e-DBT could provide comparable results to those provided by in-person therapy, allowing a more accessible version of the treatment, without sacrificing the quality of care. Additionally, both the SAQ and DERS scores significantly improved in both the e-DBT and in-person groups (*P*<.001). These results suggest that e-DBT could be an effective alternative to in-person therapy for individuals with borderline personality disorder.

Although there was no significant difference observed between the groups in terms of the number of participants who completed the program (*P*<.001), participants who prematurely terminated their involvement in the e-DBT program took part in more sessions than those who prematurely terminated participation in the in-person group. This could indicate that e-DBT offers a higher treatment adherence in individuals with borderline personality disorder.

### Limitations

Despite the strengths of this study, there are some limitations. The study did not assess the long-term efficacy of the treatment; future research should investigate this by implementing a follow-up component.

Additionally, among the participants who selected the e-DBT and in-person groups, only 44% (23/52) and 49% (27/55), respectively, completed the program. The large number of dropouts could affect the result of the study; however, we believe that the lack of adherence with treatment could be due to the nature of borderline personality disorder. For individuals with borderline personality disorder, the dropout rates in a DBT outpatient group are typically quite high, often peaking between 24% and 58% and are attributed to a younger age, higher levels of baseline distress, and a higher level of baseline nonacceptance of emotional responses [22]; therefore, the dropout rates in our study are not unusual

### Future Direction

Although the therapy at the Personality Disorder Services at Queen’s University, Kingston, Ontario is offered in 3 phases—DBT-informed skill-building group (Managing Powerful Emotions), psychotherapy groups, Chrysalis Program—in this study, electronic delivery was only offered for the first phase. Future research should evaluate the efficacy of electronic delivery of all 3 phases.

Future research is necessary to address the abovementioned limitations and to provide further support for the efficacy of an e-DBT program. Although the results of our study suggest that email is a viable method for delivering DBT skill-building groups, a randomized controlled study should be conducted to compare the efficacy of in-person DBT with e-DBT for borderline personality disorder treatment. A control group should be utilized to examine its efficacy in comparison to other delivery methods. Moreover, a benefit to randomization would be that individuals with differing technology comfort levels would be more evenly dispersed. Future research should also implement a follow-up period to ensure that e-DBT has long-term efficacy.

### Conclusions

Notwithstanding its limitations, our study’s findings have significant practical implications. This study provides evidence that DBT delivered via email can be effective in reducing the severity of symptoms associated with borderline personality disorder. These findings concurrently add to literature on the efficacy of internet-based interventions for DBT, with more work needing to be done [23]. This innovative modality has the potential to increase the accessibility of mental health services for a large group of individuals who could benefit from these resources. This simple, innovative, and user-friendly way to deliver DBT can be used to deal with barriers to treatment such as lack of resources, work or school commitments, transportation limitations, geographical isolation, the stigma associated with mental health treatment, and the high costs of software development. This treatment modality can be particularly beneficial for those comfortable with technology who may be concerned with the stigma associated with attending in-person DBT or group treatments by allowing treatment to be completed at any time and location. The DBT delivered via email shows promise as a new treatment delivery modality that can provide increased accessibility while offering improvements in symptomology that are comparable to of in-person DBT for individuals with borderline personality disorder.

## Abbreviations

DBT: dialectical behavioral therapy
DERS: Difficulties in Emotion Regulation Scale
e-CBT: electronically delivered cognitive behavioral therapy
e-DBT: electronically delivered dialectical behavioral therapy
SAQ: Self-Assessment Questionnaire
